# Up-regulation of FGF15/19 signaling promotes hepatocellular carcinoma in the background of fatty liver

**DOI:** 10.1186/s13046-018-0781-8

**Published:** 2018-07-04

**Authors:** Guozhen Cui, Robert C. Martin, Hang Jin, Xingkai Liu, Harshul Pandit, Hengjun Zhao, Lu Cai, Ping Zhang, Wei Li, Yan Li

**Affiliations:** 1 Department of Hepatology, Cancer Center, The First Hospital of Jilin University, No. 71. Xinmin Street, Changchun, 130021 Jilin China; 2 Division of Surgical Oncology, Department of Surgery, School of Medicine, University of Louisville, 511 S Floyd ST MDR Bldg Rm326A, Louisville, KY 40202 USA; 3 Department of Neurology, Neuroscience Center, The First Hospital of Jilin University, Changchun, 130021 China; 4 Department of Hepatobiliary and Pancreatic Surgery, The First Hospital of Jilin University, Changchun, 130021 China; 5 Department of Pediatrics, Kosair Children’s Hospital Research Institute, University of Louisville, Louisville, KY 40202 USA

**Keywords:** Fibroblast growth factor 15/19, Steatohepatitis, Hepatocellular carcinoma

## Abstract

**Background:**

Upregulated fibroblast growth factor 19 (FGF19) expression in human hepatocellular carcinoma (HCC) specimens is associated with tumor progression and poor prognosis. Nonalcoholic steatohepatitis (NASH) patients are at high risk for malignant transformation into HCC.

**Methods:**

A steatohepatitis-HCC model was established in male C57L/J mice treated with N-nitrosodiethylamine (DEN) and high-fat diet (HFD). A mouse HCC cell line (Hepa1–6) and a mouse hepatocyte line (FL83B) were used to elucidate the mechanism by free fatty acids (FFA) treatment. FGF15, the mouse orthologue of FGF19, and it receptor fibroblast growth factor receptor4 (FGFR4) as well as co-receptor β-klotho were studied. FGF19 signaling was also studied in human samples of HCC with steatohepatitis.

**Results:**

HCC incidence and tumor volume were significantly increased in the DEN+HFD group compared to that in the DEN+control diet (CD) group. Increased levels of FGF15/FGFR4/β-klotho, aberrant epithelial–mesenchymal transition (EMT) and Wnt/β-catenin signaling were detected in DEN+HFD mice. Blockage of the FGF15 signal can attenuate cell migration ability and aberrant EMT and Wnt/β-catenin signaling.

**Conclusions:**

Up-regulated FGF15/FGFR4 signaling promoted the development of HCC by activation of EMT and Wnt/β-catenin signaling in the lipid metabolic disorder microenvironment. Further investigation of FGF19/FGFR4 signaling is important for potential early diagnosis and therapeutic targeting in HCC patients.

**Electronic supplementary material:**

The online version of this article (10.1186/s13046-018-0781-8) contains supplementary material, which is available to authorized users.

## Novelty and impact

FGF15/19 is well studied in NASH and HCC, but it is unknown if FGF15/19 can bridge NASH-associated HCC, which has not been addressed yet. In extension of our previous study (Oncotarget. 2016;7(32):52329–52,339), FGF15/19 was further demonstrated to provide key critical signaling during the carcinogenetic transformation to HCC in a NASH-HCC mouse model. Our data demonstrated that up-regulated FGF15/FGFR4 signaling promoted the development of HCC by active CSCs signaling in the metabolic disorder microenvironment.

## Background

Upregulated FGF19 expression in human HCC specimens was found to be associated with tumor progression and poor prognosis [1]. In our previous study, we found that FGF19 was significantly increased in both serum and tumor tissue of HCC patients. Interestingly, up-regulated FGF19 signaling significantly correlated with epithelial cell adhesion molecule (EpCAM), one of biomarkers of EMT and stemness, following the steatosis-steatohepatitis-cirrhosis-HCC sequence [2]. FGF19 is an important player in postprandial gut-liver communications [3] and functions as a growth factor for hepatocytes [4, 5]. Recent evidence indicates that FGF19 gene amplification is shown to act as a driver for adult HCC [6].

FGF15, the mouse orthologue of FGF19, was first identified as a downstream target of the chimeric homeodomain oncoprotein E2A-PBX1 (pre-B cell leukemia transcription factor 1) in mice [7]. FGF19 was identified at the same time in a screen for novel FGFs in the fetal brain [8], but it was found later as the human orthologue of mouse FGF15 [9]. Following identification, their roles in metabolism were further studied. FGF15/19 is expressed abundantly in the distal small intestine; once secreted, it binds to its preferred receptor FGFR4 and co-receptor β-klotho, triggering a signaling cascade involving hepatic bile acid, lipid and glucose metabolism [10, 11]. Presently, the carcinogenetic role of FGF15/19 signaling has been recognized in various cancers, including breast, gastric, lung, prostate, nasopharyngeal carcinoma and liver cancer [12].

The incidence of malignant tumors worldwide, released in 2015, indicates that liver cancer remains the 5th and 9th in the incidence, and 2nd and 6th in the mortality for males and females, respectively [13]. HCC, as the fastest growing cause of cancer-related death, is partly attributable to the rising prevalence of non-alcoholic fatty liver disease (NAFLD) and diabetes. With an estimated prevalence of 25% globally [14], NAFLD is accepted as a common etiology for chronic liver disease. NAFLD encompasses a broad spectrum of conditions, ranging from non-progressive bland steatosis to nonalcoholic steatohepatitis (NASH), cirrhosis, and malignant transformation into HCC [15, 16]. A recent study has shown that NAFLD progresses to the progressive form-NASH in 44%, even in the patients without histological inflammation at baseline [17]. The estimated annual HCC incidence in NASH is about 0.3% [16]. Of interest is that HCC has been increasingly recognized in NASH patients without cirrhosis [18, 19]. However, it is less clear whether NASH is a required signaling for HCC carcinogenesis.

FGF19 has shown beneficial effects on the obese metabolic profile (i.e., increased energy expenditure and weight loss after chronic treatment, improved lipid profile) mediated via white adipose tissue metabolism [20], but the stimulation of proliferation by FGF19 in liver currently prevents its use to treat metabolic diseases [21]. Interestingly, knockout of FGFR4, the specific FGF19 receptor, can protect against HFD-induced hepatic steatosis, implying that the FGF19 signaling in the liver is indispensable for whole body lipid metabolism [22]. Because of the roles of FGF19 signaling in lipid metabolism and carcinogenesis, it is truly important to understand the mechanism(s) regarding the pathogenesis of NASH contributing to carcinogenetic progression. In this study, we investigated the FGF15 signaling in potential carcinogenetic transformation of HCC in a NASH-HCC mouse model using a combination of DEN and HFD in mice. The pathology of steatohepatitis and the carcinogenetic transformation of HCC as well as parameters of inflammation, lipid metabolism, cellular events, EMT and Wnt/β-catenin signaling were investigated in the mouse liver tissue. In addition, the mechanism of FFA/FGF15 signaling contributing to HCC carcinogenesis was studied in mouse hepatoma and hepatocyte cell lines. The associated biomarkers from animal model were further investigated in human HCC specimens.

## Methods

### NASH-HCC model

Sexually matured male and female C57 L/J mice (Jackson Laboratory, Maine) were set as a mating pair for breeding. The animals were housed four per cage, given indicated chow and tap water, and maintained at 22 °C and on a 12-h light/dark cycle. Male littermates of C57 L/J male mice, yielding the F1 generation, at 15 days of age received either N-nitrosodiethylamine (DEN) (Sigma, St. Louis, MO) at 40 mg/kg, or the same amount of saline as control by intraperitoneal injection (i.p.). When mice were 4 weeks old, both DEN and saline administrated mice assigned randomly into 2 groups: high fat diet (HFD) (Rodent Diet with 60% kcal% fat, D12492, Research Diets, Inc., New Brunswick, NJ) and control diet (CD) (Rodent Diet with 10% kcal% fat (D12450B, Research Diets, Inc., New Brunswick, NJ). Tumor nodules in the liver were monitored by ultrasound using high-resolution ultrasound (Vevo 770™-120 image system, VisualSonics Inc. Canada) to quantify changes in tumor growth in the liver. Body weight, glucose, glucose tolerance test (GTT) and insulin tolerance test (ITT) were recorded during the experimental period. Serum glucose assay was performed using a Sigma assay kit (Sigma-Aldrich Company, MI). Suggested by the ultrasound finding of HCC nodules, the mice in each group were sacrificed at month 2, month 6 and month 10, respectively. Serum plasma and hepatic tissues were harvested for further analysis. Serum plasma alanine aminotransferase (ALT) and alpha-Fetoprotein (AFP) were measured using an ALT infinity enzymatic assay kit (Thermo Fisher Scientific Inc., Waltham, MA) and mouse AFP Quantikine ELISA Kit (R&D Systems, Inc. Minneapolis, MN). The animal procedures were approved by the Institutional Animal Care and Use Committee of University of Louisville, which is certified by the American Association for Accreditation of Laboratory Animal Care.

### Gross anatomy and histopathological examination

At respective time points, the animals were sacrificed and whole livers were isolated and weighted. The isolated livers were examined macroscopically for tumor growth, and numbers of foci nodules were counted and recorded for each animal. The length and width of the tumor nodule was also measured. The harvested tissues were fixed in 10% neutral phosphate buffered formalin or embedded in Optimal Cutting Temperature medium (OCT) and frozen in liquid nitrogen. The formalin fixed tissues were further embedded in paraffin and sectioned to a thickness of 5 μm for histological and immunohistochemical examinations. Oil Red O staining for lipid accumulation in the liver tissues was performed in OCT-embedded frozen tissue. Hematoxylin-and-eosin (H&E) staining for histology was performed in paraffin-embedded frozen tissue. The images were reviewed and analyzed microscopically for determination of NASH and/or HCC.

### Immunohistochemical (IHC) analysis

Immunohistochemical staining was performed on the paraffin-embedded tissue sections. Endogenous peroxidase was blocked with 3% hydrogen peroxide, and then with 5% animal serum for 30 min to block non-specific reactions. These tissue sections were incubated with primary antibodies (see antibody list in Additional file 1). Tissue sections were incubated with horseradish peroxidase-conjugated secondary antibodies (1, 300–400 dilutions with PBS) for 2 h at room temperature, and then incubated with peroxidase substrate DAB kit (Vector Laboratories, Inc., Burlingame, CA) to develop brown color. The counterstaining was performed using hematoxylin or methyl green. A negative controls without primary antibodies was included in each run. Triple staining was performed on human sample using a Triplestain IHC Kit (Abcam, ab183286, Cambridge, MA) according to the provided factory instructions. Digital images were acquired with the Olympus 1 × 51 microscope (Olympus, Pittsburgh, PA) at 10× magnification using the Olympus DP72 digital camera and the length of scratch-wound was measured via the cellSens Dimention imaging system.

### Western blot assay

The protein levels for the biomarkers were semi-quantified by Western blot analysis as described previously [2]. Electrophoresis was performed on 12% SDS-PAGE gel and the proteins were transformed to nitrocellulose membrane. The membranes were incubated with the primary antibodies (see antibody list in Additional file 1: Table S1) overnight at 4^°^C and with secondary antibody for 1 h at room temperature. The antigen-antibody complexes were then visualized using ECL kit (Amersham, Piscataway, NJ). The protein bands were quantified by densitometry analysis.

### Real-time RT-PCR (qPCR)

Total RNA was extracted using the TRIzol reagent (Invitrogen). First-strand complimentary DNA (cDNA) was synthesized from total RNA, according to manufacturer’s protocol from the RNA PCR kit (Promega, Madison, WI, USA). Quantitative PCR was carried out using the ABI 7300 real-time PCR system (Applied Biosystems, Carlsbad, CA). The primers are listed in the Additional file 1. The target mRNA expression was quantified and β-actin was used as an endogenous reference. For the list of primers, see Additional file 1: Table S2. Results were expressed as fold change in gene expression.

### Flow cytometry

The cells were suspended in PBS at a concentration of 1 × 10^6^ cells/ml. After washing with an ice-cold incubation buffer, the cells were fixed with a freshly prepared 4% paraformaldehyde solution in PBS at 37 °C. For primary antibody incubation, cells were washed twice with incubation buffer and resuspended in primary antibodies diluted in incubation buffer for 1 h at room temperature. After completion of all staining steps, the cells were washed with incubation buffer and resuspended in 500ul incubation buffer. The samples were evaluated with a BD FACS Canto II flow cytometer (BD Biosciences). FlowJo 7.6.1 software was used for the data analysis.

### Cell lines and in vitro study

Cell lines including a mouse hepatoma cell line Hepal-6 (ATCC CRL-1830), a mouse hepatic cell line FL83B (ATCC CRL-2390), HepG2 (ATCC HB-8065), a human HCC cell line, and H4IIE (ATCC CRL1548), a rat hepatoma cell line, were used for in vitro study. Hepal-6, HepG2 and H4IIE cells were cultured in DMEM medium supplemented with 10–15% fetal bovine serum. FL83B cells were cultured in the F12 K medium (ATCC) supplied with 10% fetal bovine serum. To study the effects of FFA on the cell lines regarding the FGF15 signaling, palmitate (PA) media (Sigma, P9767) was made by dissolving 2% bovine serum albumin (BSA, US Biologicals, A1311) in serum-free DMEM supplemented with 1% penicillin/streptomycin. The treatment medium was prepared from a high concentration (40 mM) stock palmitate solution made by dH_2_O heated to 70 °C. An aliquot from the stock was then added drop wise to 2% BSA medium with constant stirring to achieve the desired final palmitate concentration. Hapal-6 cell, H4IIE cell and HepG2 cell were treated with FFA and cultured for 0, 24, 48 and 72 h. At respective time points, the culture supernatant was collected, centrifuged and filtered through a 0.45 μm filter (Millipore) to determine the secretion levels of FGF19 and FGF15 by ELISA assays. Chemical inhibitors included BLU9931 (Calbiochem, Cat#: 5.38776.0001) and (Calbiochem, Cat#: 575545-10MG). BLU9931 is the first selective small molecule inhibitor of FGFR4 with IC50 of 3 nM, while XAV939 is a cell-permeable dihydrothiopyranopyrimidinol that binds TNKS1/PARP5a and TNKS2/PARP5b to inhibit their PARsylation (poly ADP-ribosylation) activity (IC50 = 11 and 4 nM, respectively). XAV939 was used as Wnt inhibitor because it suppressed cellular axin1/2 PARsylation and ubiquitination/proteasomal degradation, resulting in axin build-up, β-catenin destruction, and blockage of the Wnt signaling. For inhibition experiments, BLU9931 was applied at the concentration of 100 nM and XAV939 was applied at the concentration of 100 μM, up to 24 h treatments based on the previous report [23, 24].

### ELISA assays

The protein levels of FGF19 were determined using a FGF19 ELISA assay kit (R&D Systems, Inc. Minneapolis, MN) according to the manufacturer’s instructions as previous report [2]. In brief, the samples were standardized corresponding to the total protein concentration. The FGF-19 standards were reconstituted with 1.0 mL of deionized water. Assay Diluent RD1S was added into each well of the provided 96-well plate. Then, standard and sample were added and incubated for 2 h at room temperature. After washing, 200 μL of FGF-19 conjugate was added and incubated for 2 h at room temperature. Substrate Solution was added for 30 min at room temperature, and stopped by Stop Solution. Optical density (OD) was determined using a microplate reader at 450 nm. FGF15 protein levels were detected by an ELISA assay, modified from previous reports [25, 26]. In brief, 100 μL dilution of FGF15 protein standards (0–2 ng/ml) and samples with capture antibody was coated in 96 well microplate overnight at 4 °C using a DuoSet ELISA Ancillary Reagent kit 2 (R&D Systems, Inc. Minneapolis, MN). After washing, the coated wells were adding 200 μL blocking buffer (1% BSA) for at least 2 h at room temperature. One hundred microliter anti-FGF15 goat IgG (AF6755) in Reagent Diluent (50 ng/ml) was applied overnight at 4 °C, and then 100 μL donkey anti-goat IgG-HRP in Reagent Diluent (200 ng/ml) was applied 2 h at room temperature Substrate Solution was added for 30 min at room temperature, and stopped by Stop Solution. The OD value of each well was read using a plate reader at 450 nm. Concentration of the FGF15/19 was analyzed based on standard curve.

### Cell migration assay

For cell migration assays, cells were collected by trypsinization following 48 h culture in FFA or BSA media with serum. The treated cells were washed with PBS, and neutralized with regular DMEM media. The cells were then counted and seeded on BD Biocoat 8 μm membrane inserts (BD Biosciences, 354,480) to perform the migration assay. The inserts were placed in wells containing regular DMEM media containing 10% FBS as chemoattractant. After 24 h, the inserts were removed, washed with PBS, fixed in methanol and stained with crystal violet (0.05% *w*/*v* in methanol). The bottom surfaces of the stained inserts were then observed under a light microscope, and the numbers of stained cells were counted in 5 fields/insert. The cell migration capacity was calculated based on the numbers of stained cells.

### Scratch-wound healing assay

For scratch-wound healing assays, cells were seeded in 24-well plates with regular media. After a 24-h acclimatization period, the cells were washed with PBS and cultured for 48 h in FFA or BSA media. Following the 5-day incubation, cells were washed with PBS. Using the tip of a sterile 10 uL pipette, a single scratch was made on the cell surface within each well. Following the scratch for 24 h and 48 h, digital images were acquired with the Olympus 1 × 51 microscope (Olympus, Pittsburgh, PA) at 10× magnification using the Olympus DP72 digital camera. Three standardized bright-field images were recorded for each scratch at the 0, 24, and 48-h time points. The length of scratch-wound was measured via the cellSens Dimention imaging system.

### RNA interference assay

Small interfering RNA (siRNA) oligos specific targeting to mouse CD36 and peroxisome proliferator-activated receptor α (PPAR-α) (siPPARa Cat #: 151210; siCD36 Cat #: 160081] and specific targeting to human CD36 and PPAR-α (siCD36 Cat #: s2645; siPPARa Cat #: s10880) as well as a scrambled negative control siRNA (Cat # 4390849) were designed and synthesized by LifeTechnologies (CA, USA) Hepl-6 and HepG2 cells were seeded at 4 × 10^5^ cells per well in a 6-well plate. Transfection was performed using Lipofectamine RNAiMAX (LifeTechnologies, CA, USA) according to the instructions of the manufacturer. A total of 100 pmol/well of siRNA was used for each 6-well plate transfection. After an 48-h transfection period, the wells were exposed to FFA for Western blot analysis.

### Human HCC tumor samples

The human HCC samples were prospectively collected from 33 patients who had undergone liver resection for hepatocellular carcinoma between 2002 and 2014. Tumor tissues along with corresponding adjacent benign tissues from the same HCC patients were acquired from the James Graham Brown Cancer Center Bio-Repository at the University of Louisville following an IRB-approved protocol. A microscope examination of the cellular composition of hepatic tissue confirmed the diagnoses of adjacent benign tissues, steatohepatitis and HCC on these liver tissues reviewed by two pathologists independently, blinded to the subject’s clinical history. The classification of adjacent benign tissues was assigned if there were no abnormalities such as cancerous cells, steatosis, fibrosis, necrosis, inflammatory infiltration, nor lipid drops found in the tissue. The specimen was designated as steatohepatitis if there was an infiltration or considerable lipid deposition with additional inflammatory changes. HCC was the designated histology if there were cancerous cells. This study was approved by the Institutional Review Board for Human Study at the University of Louisville.

### Statistical analysis

Data were collected from repeated experiments and were presented as mean ± SD. One-way ANOVA was used to determine if a difference exists. If so, a post hoc Turkey’s test was used for analysis of the difference between groups, with Origin 8 laboratory data analysis and graphing software.

## Results

### HFD accelerated and worsened the DEN-induced tumor growth in mice

**Fig. 1:**
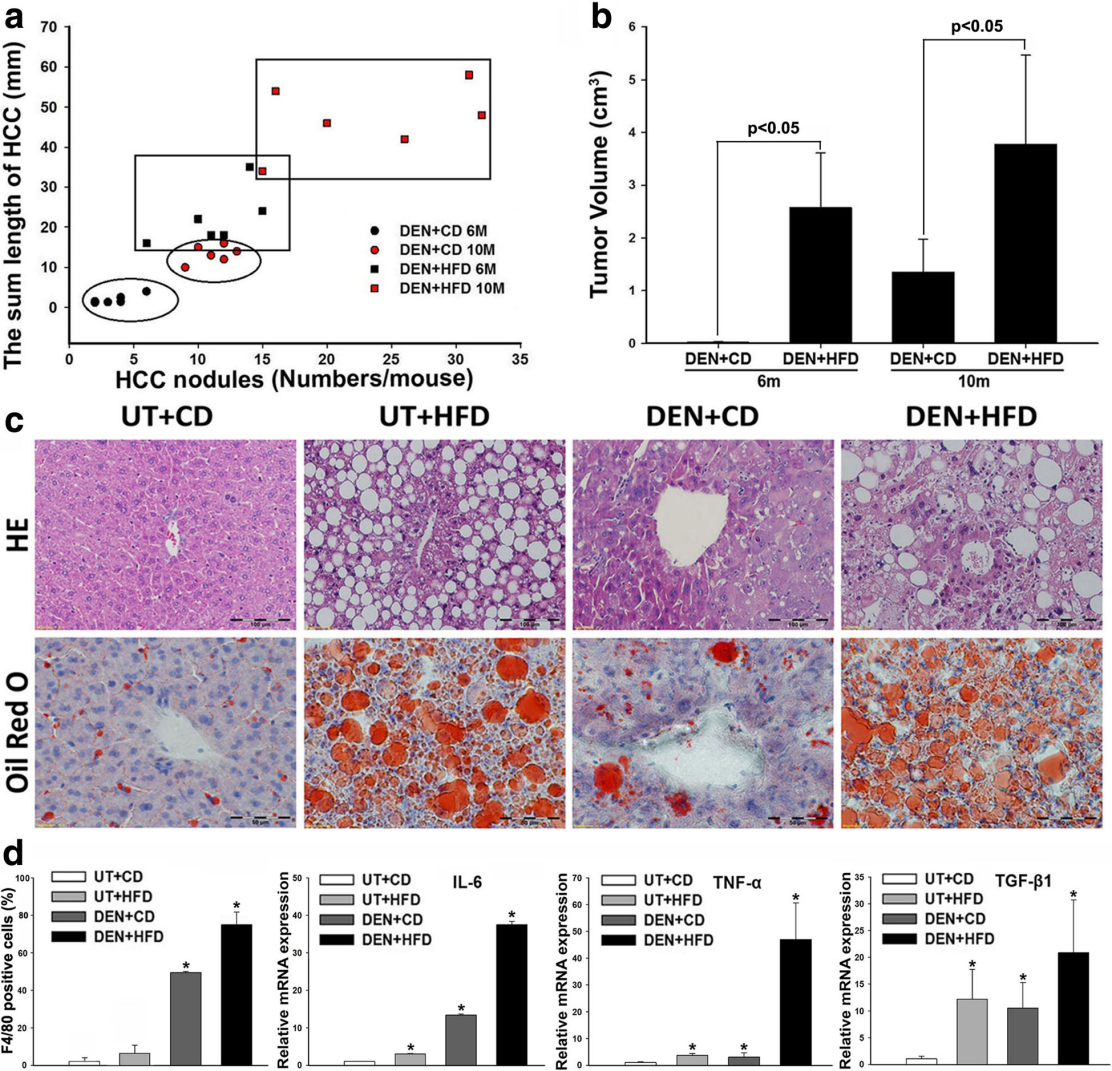
**a** The HCC tumor growth pattern represented as the regression of HCC nodule length and HCC nodule numbers in individual HCC mouse. **b** Tumor volume from 4 groups at month 6 and month 10. **c** Representative histological changes from all 4 experimental groups at month 10. Histological changes of NASH-HCC in DEN+HFD mice were identified in tissue sections by H&E staining. Lipid drops were identified in tissue sections by Oil red O staining. NASH was found typically affecting the liver parenchyma with macrovesicular changes presented in a predominantly perivenular distribution. In addition to fatty change, many hepatocytes in acinar showed ballooning and contain Mallory’s hyaline, and mixed infiltrate of inflammatory cells. UT: untreated; CD: control diet; HFD: high fat diet; DEN: N-nitrosodiethylamine. **d** Positive F4/80 cells and mRNA expressions (IL-6, TNF-α and TGF-β1) in the liver tissues from 4 groups at month 6 and month 10. *, *P* < 0.05 vs UT + CD

Ultrasound was performed to monitor the tumor growth in the mice monthly. No tumor mass was detected by ultrasound in all the groups at month 2. Tumor nodule(s) were detected in one mouse at month 3, three mice at month 4, and all six mice at month 6 from DEN+HFD group. While in DEN+CD group, tumor nodule was detected in one mouse at month 6, two mice at month 7, five mice at month 8, and all six mice at month 10. Suggested by the ultrasound findings, the mice were sacrificed at month 2 (no tumor detection), at month 6 (tumor detected in all mice of DEN+HFD group) and at month 10 (tumor detected in all mice of DEN+CD group). The control groups of untreated (UT) + CD and UT + HFD were also sacrificed at same time points. The gross anatomy confirmed the ultrasound findings in which there was no HCC nodule found on the livers of DEN+HFD and DEN+CD treated mice from month 2, but HCC nodules were found in those two groups from 6 and 10 months (Additional file 2: Figure S1). Macroscopically, the length and number of HCC nodules were calculated in all HCC mice. The results indicated that the DEN+HFD mice showed an aggressive tumor growth pattern; the length and number of HCC nodules were significantly increased compared to the same age DEN+CD mice (Fig. 1a). The right and left lobes from individual livers were sliced sagittally into 3 pieces. Tumor volume was further calculated based on the length and width of tumors on the sagittal panels. The results of tumor volumes were consistent with the result calculated by length and number of tumor nodules (Fig. 1b). Histological examination was further performed in the micro-section with H&E staining in all the mice. Bland steatosis was characterized as wildly distributed lipid drops being detected in UT + HFD mice, while steatohepatitis was characterized as infiltration of inflammatory cells in the acinar zone and in the form of hepatocyte ballooning being detected in DEN+HFD mice (Fig. 1c). The tumor nodules were confirmed as HCC foci by the cytological features of cancerous cells ranging from well to poorly differentiated distributed in parenchyma showing an abnormal hepatic architecture (Fig. 1c). The characteristics of steatohepatitis and HCC in DEN+HFD mice indicated the success of NASH-HCC model, which was further supported and confirmed by microphage infiltration and up-regulated expression of pro-inflammatory cytokines (Fig. 1d). The hepatic lipid accumulation was further confirmed by Oil Red O staining in hepatic and cancerous tissue. As shown in the Fig. 1c, the wide distribution of positive-stained large lipid drops was found in the UT + HFD mice and DEN+HFD mice. Interestingly, the DEN+CD mice at month 10 also showed positive staining of lipid drops, implying aberrant lipid metabolism during the HCC initiation and development.

### Increased FGF15 in NASH-HCC mice with DEN+HFD treatment

**Fig. 2:**
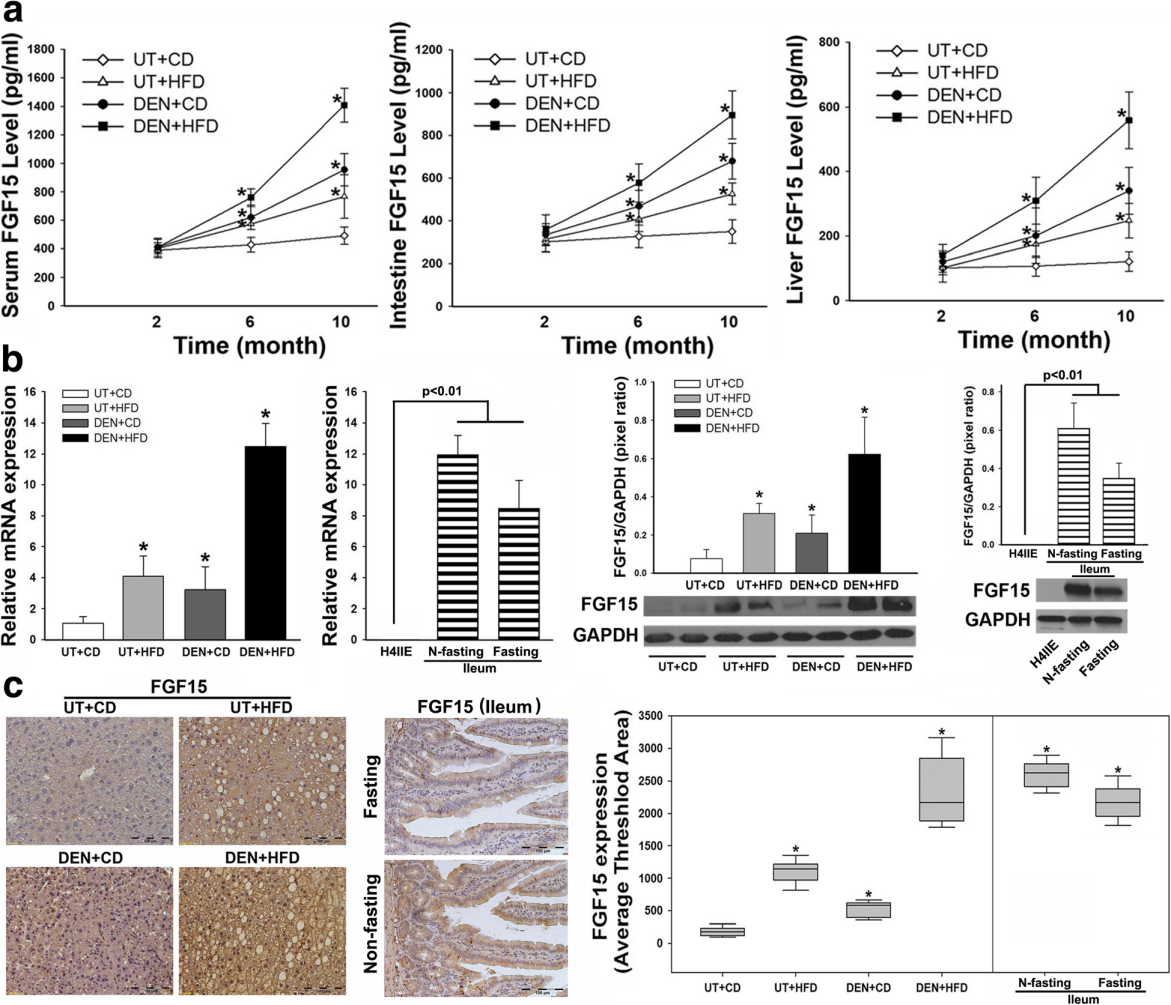
**a** The protein levels of FGF15 in serum, ileum and liver tissue by ELISA assays. **b** the FGF15 mRNA levels and protein levels from 4 groups at month 10. The lysates of ileal tissues from fasting and non-fasting wild type mice are used as positive controls for FGF15, while the lysate of H4IIE cells, a rat hepatoma cell line, is used as a negative control because rats do not express FGF15. N-fasting: non-fasting. **c** Representative images of FGF15 protein distribution in hepatic parenchyma and computer image-quantification in all 4 experimental groups at month 10 by immunohistochemical staining. Ileal tissues from fasting and non-fasting wild type mice are used as positive controls for FGF15 IHC staining. N-fasting: non-fasting. UT: untreated; CD: control diet; HFD: high fat diet; DEN: N-nitrosodiethylamine. *, P < 0.05 vs UT + CD

Because the aggressive pattern of tumor growth in DEN+HFD mice was identified, metabolic disorder may play a critical role in contributing to tumor growth. Therefore, parameters of metabolic disorder contributing to steatohepatitis were further evaluated. Significantly increased body weight, liver weight and TG level in hepatic tissue was found in DEN+HFD mice at 6 months when HCC developed in all mice, implying that lipid metabolic disorder might accelerate the DEN-induced carcinogenetic transformation. In addition, impaired glucose tolerance and insulin tolerance were also observed in the DEN+HFD mice. Consistent with aberrant lipid metabolism, significantly increased serum ALT and alpha-fetoprotein (AFP) was found in DEN+HFD mice (Additional file 3: Figure S2), confirming that repetitive damage of hepatocytes contributed to carcinogenetic transformation. Aggressive growth of HCC and metabolic abnormalities in the DEN+HFD mice led us to analyze further FGF15 because of its metabolic action and tumorigenic role. Significantly, an increased FGF15 level in blood was found in the DEN+HFD mice compared to other groups. Because FGF15 was synthesized predominately in the ileum, protein levels of FGF15 were further determined in the ileum tissue. As expected, significantly increased protein levels of FGF15 in ileum tissue was found in DEN+HFD mice, compared to that from other groups. Hepatic FGF15 protein levels were also significantly increased in DEN+HFD mice, especially from mice with DEN+HFD treatment for 10 months, compared to the other 3 groups (Fig. 2a). In fact, increased hepatic FGF19 from HCC patients and increased hepatic FGF15 from mice were previously reported [2, 12, 27, 28]. We performed confirming studies for FGF15 expression at the mRNA level by RT-PCR and at the tissue protein level by Western blot and IHC. The lysates and total RNA of ileal tissues from fasting and non-fasting male adult C57 L/J mice were used as positive controls for FGF15 expressions of protein and mRNA. All results indicated that the mRNA levels and protein levels in liver tissues were significantly upregulated in DEN+HFD mice (Fig. 2b-c). Considering these findings, it would be critical to investigate further the FGF15 signaling for its role it may play in HCC transformation.

### Upregulation of FGF15 receptors and fatty acid synthase (FASN) signaling in DEN+HFD treated mice

**Fig. 3:**
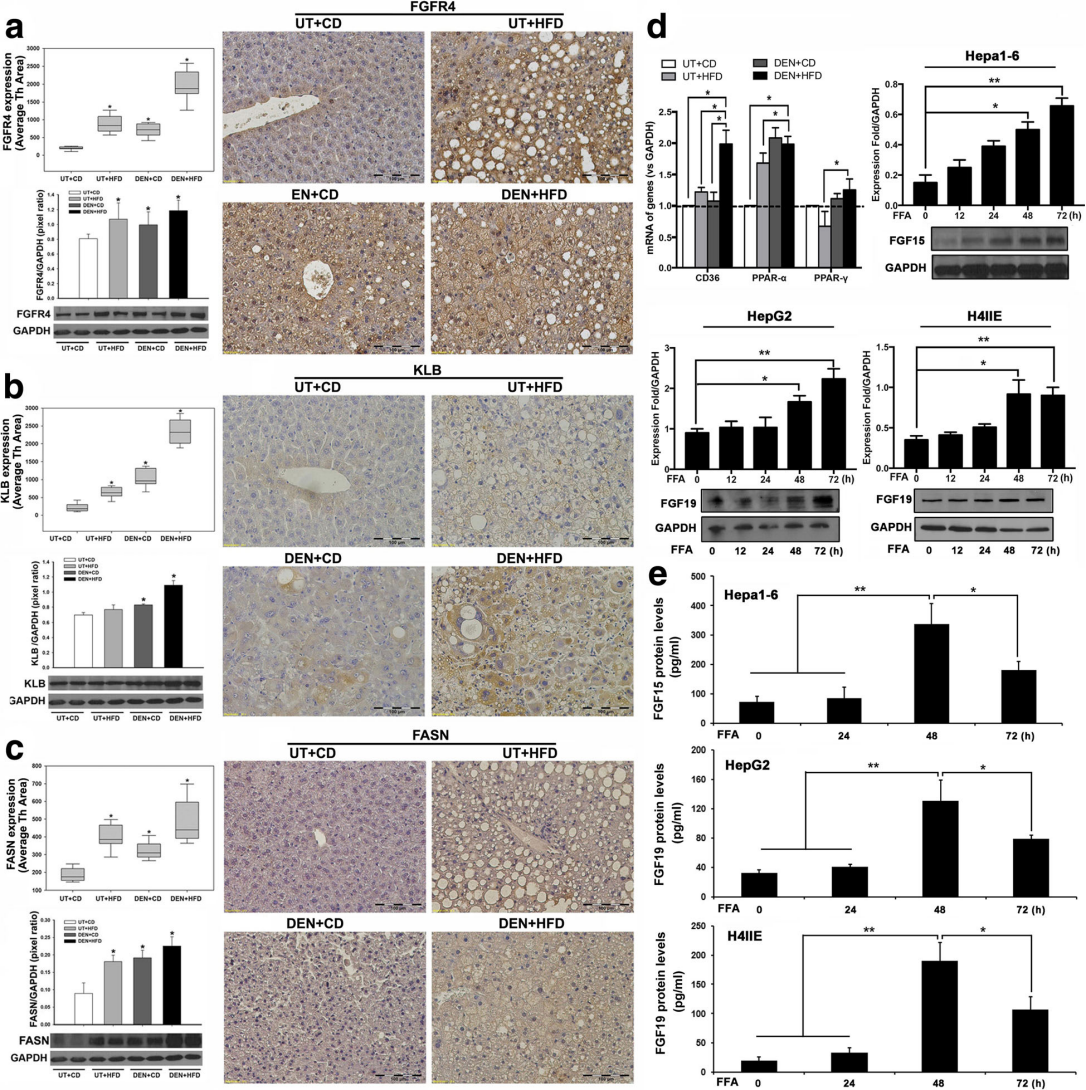
**a**-**c** Representative images of IHC for FGFR4, β-klotho, and FASN in hepatic tissues and protein expression by computer image-quantification, and Western blot analysis of protein levels in the 4 experimental groups at month 10. The protein expression by computer image-quantification presented as Average Threshold Area (Average Th Area). The protein levels of Western blot analysis were quantified by image analysis and presented as pixel ratio over the control GAPDH. KLB: β-klotho; UT: untreated; CD: control diet; HFD: high fat diet; DEN: N-nitrosodiethylamine. *, P < 0.05 vs UT + CD. **d** mRNA expressions (CD36, PPAR-α and PPAR-γ) in the liver tissues from 4 groups at month 6 and month 10. Western blot analysis of FGF15 protein levels in three HCC cell lines (Hepal-6, HepG2 and H4IIE) challenged by FFA 0–72 h. Western blot analysis were quantified by image analysis and presented as pixel ratio over the control GAPDH. **e** The protein levels of FGF15 and FGF19 in the supernatant of Hepa1–6, H4IIE, and HepG2 cells by ELISA assays. *, P < 0.05; **, *P* < 0.01

As previously mentioned, FGF15 binds to FGFR4 and co-receptor β-klotho to trigger FGF15 signaling. Aberrant signaling through the FGF15/19-FGFR4 pathway was associated with HCC development in mice [29] and with poorer prognosis [30] in HCC patients. Therefore, the FGF15 receptor FGFR4 and co-receptor β-klotho were investigated in the liver tissues. The results of IHC indicated that FGFR4 was extensively distributed in the hepatocytes, while treatments (UT + HFD, DEN+CD, and DEN+HFD) significantly increased the expression of FGFR4. The FGFR4 expression was much higher in DEN+CD mice and DEN+HFD mice than in UT + CD mice (Fig. 3a). A weak expression of β-klotho was found in the UT + CD control group, while treatments (UT + HFD, DEN+CD, and DEN+HFD) significantly increased the expression of β-klotho, especially in the DEN+HFD treatment group. The results of IHC were confirmed by Western blot, in which similar expression patterns of FGFR4 and β-klotho were found (Fig. 3b). Because FGF15-FGFR4-β-klotho signaling involved regulation of lipid metabolism, aberrant hepatic de novo lipid synthesis was closely related to the carcinogenesis. We further examined an important enzyme, fatty acid synthase (FASN), which has been reported as responsible for de novo lipogenesis and carcinogenesis [31]. The Western blot and IHC results indicated that treatments (UT + HFD, DEN+CD, and DEN+HFD) significantly increased the expression of FASN (Fig. 3c), implying that FGF15 signaling is linked to aberrant de novo lipogenesis during the HCC carcinogenetic transformation. To further study FASN downstream efforts involved in lipogenesis, the biomarkers of fatty acid signaling including CD36, PPAR-α, and PPAR-γ are evaluated. The qPCR results indicated that the mRNA levels of CD36 were significantly increased in the mice with treatment (DEN+HFD), while the mRNA levels of PPAR-α were significantly increased not only in the mice with treatment (DEN+HFD) but also with treatment (UT + HFD and DEN+CD). However, mRNA levels of PPAR-γ were not significantly increased in the DEN+HFD mice compared to UT + CD mice (Fig. 3d). To study if FGF15/19 could be induced by FFA, Hapal-6 cell (a mouse hepatoma cell line), H4IIE (a rat hepatoma cell line) and HepG2 (human HCC cell line) were tested. The Western blot results indicated that protein levels of FGF15 from Hapal-6 cells and protein levels of FGF19 from H4IIE and HepG2 cells were increased in a time-effective manner. Significantly increased FGF15 and FGF19 were found after 48 h treatment with FFA (Fig. 3d). The secretion levels of FGF19 and FGF15 were determined by ELISA assays. It was interesting to find out that the secretion levels of FGF19 and FGF15 were increased at 48 h but the increases were blunt at 72 h. This results indicated increases of harboring FGF15/19 proteins with FFA treatment at 72 h (Fig. 3e), implying that the FFA induced harboring FGF15/19 proteins might be more important for the FGFR4 activation contributing to HCC carcinogenetic transformation, as previous reported [32].

### FFA mediated EpCAM and Wnt/β-catenin signaling

**Fig. 4:**
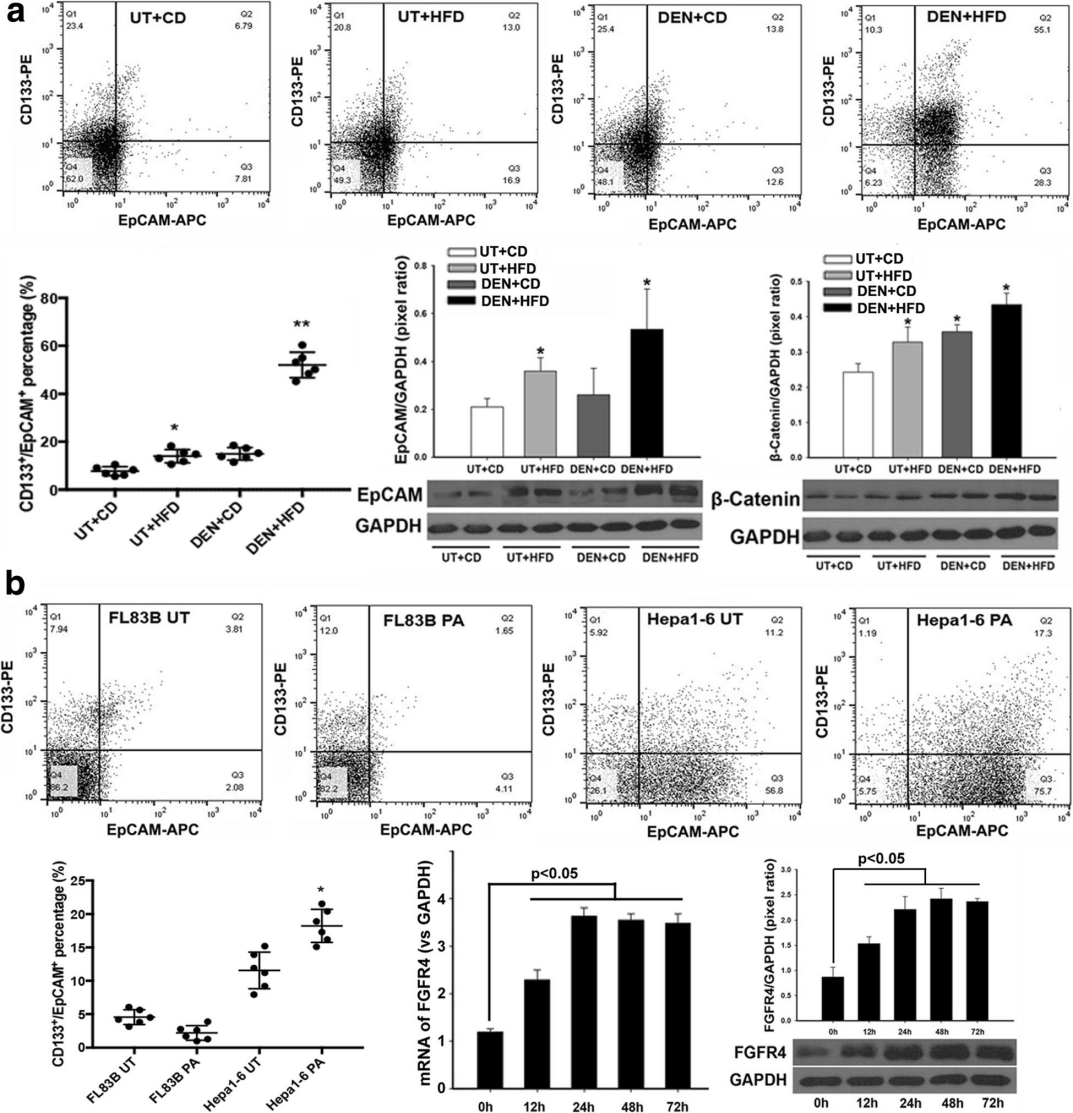
**a** EpCAM-positive and CD133-positive cells were evaluated by flow cytometry in the “benign tissues” adjacent to tumor mass as well as in non-tumor tissue. Western blot analysis of EpCAM and β-catenin protein levels in liver tissues from 4 experimental groups at month 10. **b** EpCAM-positive and CD133-positive cells were evaluated by flow cytometry in FL83B and Hepal-6 cells treated by FFA for 48 h. Time-course study of FGFR4 mRNA and protein levels by qPCR and Western blot in Hepal-6 cells challenged by FFA 0–72 h. Analysis for qPCR and Western blot was presented as the fold ratio over GAPDH control. UT: untreated; CD: control diet; HFD: high fat diet; DEN: N-nitrosodiethylamine; h: hour. *, *P* < 0.05 vs UT + CD

HCC expressing stemness markers are characterized by aggressive behavior [33]. Because EpCAM and CD133 are well-accepted surface markers in HCC [34, 35], we further analyzed the EpCAM and CD133 expression to study their potential roles during HCC carcinogenetic transformation in NASH-HCC mice. The normal hepatocytes were isolated from non-tumor liver tissues (CD mice and HFD mice) and the “hepatocytes” were isolated from the “benign tissues” adjacent to tumor mass (DEN-CD mice and DEN-HFD mice). Single cell suspension was performed using gentleMACS™ Dissociator (Miltenyi Biotec Inc., San Diego, CA) according to the product instructions. EpCAM-positive cells and CD133-positive cells were detected by flow cytometry assay. The results indicated that much higher numbers of EpCAM-positive cells from 10 months DEN+HFD mice. A possible reason could be that the cells in the tissues adjacent to the tumor mass were potentially cancerous, even though they appeared benign macroscopically and microscopically. As we observed in the HCC mice, the tumor nodules generate from multiple clones during disease development (Additional file 3: Figure S2) and the so-called “benign tissues” may be premalignant and the next tumor growth sites. The significantly increased EpCAM expression was further confirmed by Western blot (Fig. 4a). Because activation of Wnt pathway was well reported for HCC carcinogenesis, we further analyzed protein levels of β-catenin, a key component in Wnt-pathway related EpCAM. Interestingly, the results showed that significantly increased β-catenin was found not only in “benign tissues” adjacent to tumor mass (DEN-CD mice and DEN-HFD mice) but also in non-HCC tissues from the mice with HFD treatment from 10 months (Fig. 4a). These results indicated that hepatic lipid metabolism could play an important role affecting EpCAM and Wnt/β-catenin signaling-mediated carcinogenesis. Previous studies reported that the excessive hepatic accumulation from FFAs induced metabolic disorder [36, 37]. To elucidate effect of FFAs on FGF15 signaling, in vitro studies were performed using a benign hepatocyte cell line, FL83B, and a mouse hepatoma cell line, Hepal-6. Both FL83B cells and Hepal-6 cells were treated with palmitate, a critical component of FFA. Flow cytometry assay detected fewer cells (3%) with positive EpCAM and CD133 staining in the benign FL83B hepatocyte cells (Fig. 4b), indicating that FFA treatment did not significantly affect EpCAM and CD133 expression in FL83B cells, This result was consistent with the flow cytometry results from the hepatocytes isolated from the benign tissues (CD mice and HFD mice). Higher numbers of Hepal-6 cells (12%) showed positive EpCAM and CD133 staining. There was a shift in EpCAM expressing Hepa1–6 cells from 68.5 to 92.3% after FFA treatment but CD133-positive cells only showed a marginal increase from 17.1 to 18.5% (Fig. 4b), implying a critical role of FFA played on EpCAM expression. The mRNA and protein levels of FGFR4 in Hepal-6 cells challenged by FFA were further determined by qPCR and Western blot. Significantly up-regulated mRNA levels and protein levels of FGFR4 were found in the Hepal-6 cells challenged with FFA from 12 h to 72 h (Fig. 4b). The increased EpCAM-positive cells could be a potential mechanism underlying the EMT-mediated development of HCC [38]. The increased expressions of EpCAM and β-catenin were further confirmed by IHC results both in vivo and in vitro (Additional file 4: Figure S3). Taken together, the data supported the speculation that FGF15 signals played important roles in contributing to the cellular EMT-mediated HCC carcinogenesis.

### Blockage of FGFR4 abolished the FFA-promoted migration ability and aberrant signaling

**Fig. 5:**
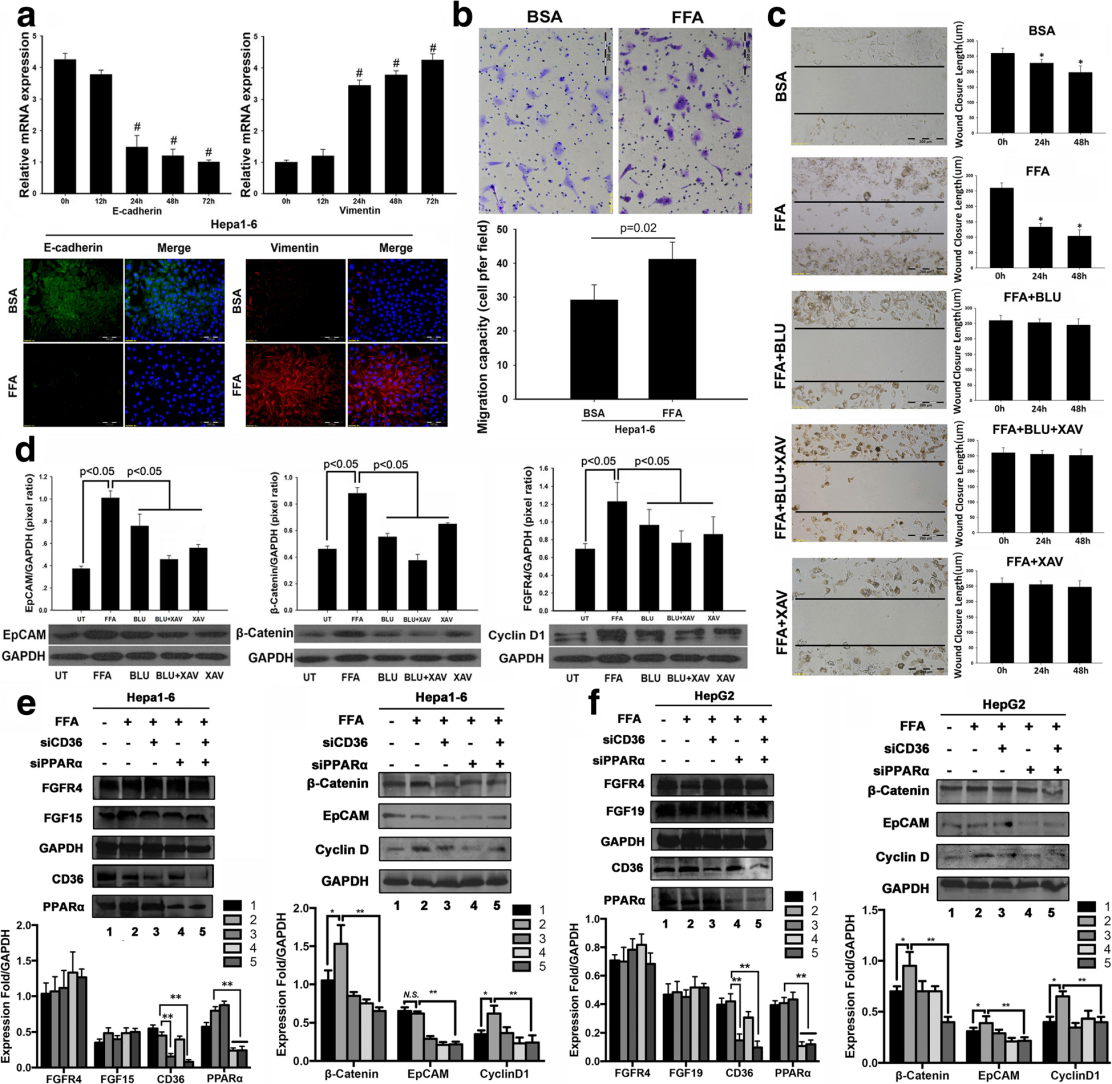
**a** Time-course study of relative mRNA levels of E-Cadherin and vimentin in hepal-1 cells treated by FFA. The representative images of E-Cadherin and vimentin in hepal-1 cells treated by FFA at 72 h. **b** A trans-well migration assay to study the migratory ability of Hepal-6 cells in response to FFA treatment. Migration cell number was calculated. **c** A scratch-wound healing assay to study the migratory ability of Hepal-6 cells in response to FFA treatment. A 250 μm scratch-wound was made on 90% confluent cells in each treatment group. Average length of scratch-wound was calculated in each treatment group and the migratory ability of cells was represented as wound closure length. *: *P* < 0.05 vs 0 h. **d** Western blot analysis of EpCAM, β-catenin, FGFR4 and FASN in hepal-1 cells treated by FFA. The protein levels were quantified by image analysis and presented as pixel ratio over the control GAPDH. ^#^: *P* < 0.05 vs 0 h. FFA: free fatty acid; h: hour. UT: untreated; BLU: BLU9931; XAV: XAV 939. **e**-**f** siPPARa and siCD36 as well as the scrambled siRNA were applied to the Hapal-6 and HepG2 cells for 48 h to silence the mRNA of PPAR-α and CD36, and the cells were challenged by FFA for 48 h. Western blot analysis was performed to determine the protein levels of FGF15, FGF19, FGFR4, EpCAM, β-catenin, andcyclin D. 1: scrambled siRNA; 2: FFA; 3: FFA+ siCD36; 4: FFA+ siPPARa; 5: FFA+ siCD36 + siPPARa. *N.S.*: no significant importance; *, P < 0.05; **, P < 0.01

We further evaluated two EMT biomarkers, E-cadherin and vimentin, in Hepal-6 cells treated with FFA. Decreased E-cadherin expression and increased vimentin expression were found, indicating loss of epithelial characteristics and acquisition of mesenchymal phenotypes in Hepal-6 cells with FFA treatment (Fig. 5a). EMT is a complex process which enhances cancer cell motility and is thought to play an important role in tumor-initiating [39]. Switching from epithelial phenotype to mesenchymal phenotype, a motile phenotype encouraged us to further investigate the motility of Hepal-6 cells by FFA treatment. A trans-well migration assay was performed to study the migratory ability of Hepal-6 cells in response to FFA treatment. As expected, the migration ability of Hepal-6 cells was significantly increased by FFA treatment compared to the BSA treatment control (Fig. 5b). Using the inhibitor BLU9931 to block FGFR4 and stabilizing axin by XAV939 to stimulate β-catenin degradation, thereby blocking Wnt/β-catenin signalling, cell motility was further studied by a wound-healing assay. With blockages of FGFR4, Wnt/β-catenin signaling, or a combination of both signals, the FFA-induced migration ability of Hepal-6 cells was abolished (Fig. 5c). To study if FGF15 signaling was linked to EpCAM-Wnt/β-catenin and a down-stream target of cell growth, expressions of EpCAM, β-catenin, and CyclinD1 were further investigated using the above two inhibitors in Hepal-6 cells challenged by FFA. Western blot analysis indicated that inhibition of FGFR4 dramatically decreased the FFA induced EpCAM, β-catenin and cyclinD1 (Fig. 5d). Inhibition of Wnt/β-catenin signaling showed similar result to the FGFR4 inhibitor. To study whether silencing the key genes in FFA signaling could affect these downstream molecular events, siPPARa and siCD36 were applied to the Hapal-6 and HepG2 cells to silence the mRNA of PPAR-α and CD36, and then the cells were challenged by FFA. The results showed that silencing the mRNA caused significant decreases of the PPAR-α and CD36 protein productions. siRNA knockdown of PPAR-α and CD36 significantly attenuated the FFA induced increases of EpCAM, β-catenin and cyclinD1, but no obvious changes of FGF15/19 and FGFR4 protein levels and the cell survival were observed (Fig. 5e-f). Taken together, a crosstalk between FFA and FGF15/19-FGFR4 signaling could exit and play a critical role in contributing to tumor-initiation and the development of HCC.

### Aberrant signaling of FGF19/FGFR4/ β-klotho in human NASH-HCC

**Fig. 6:**
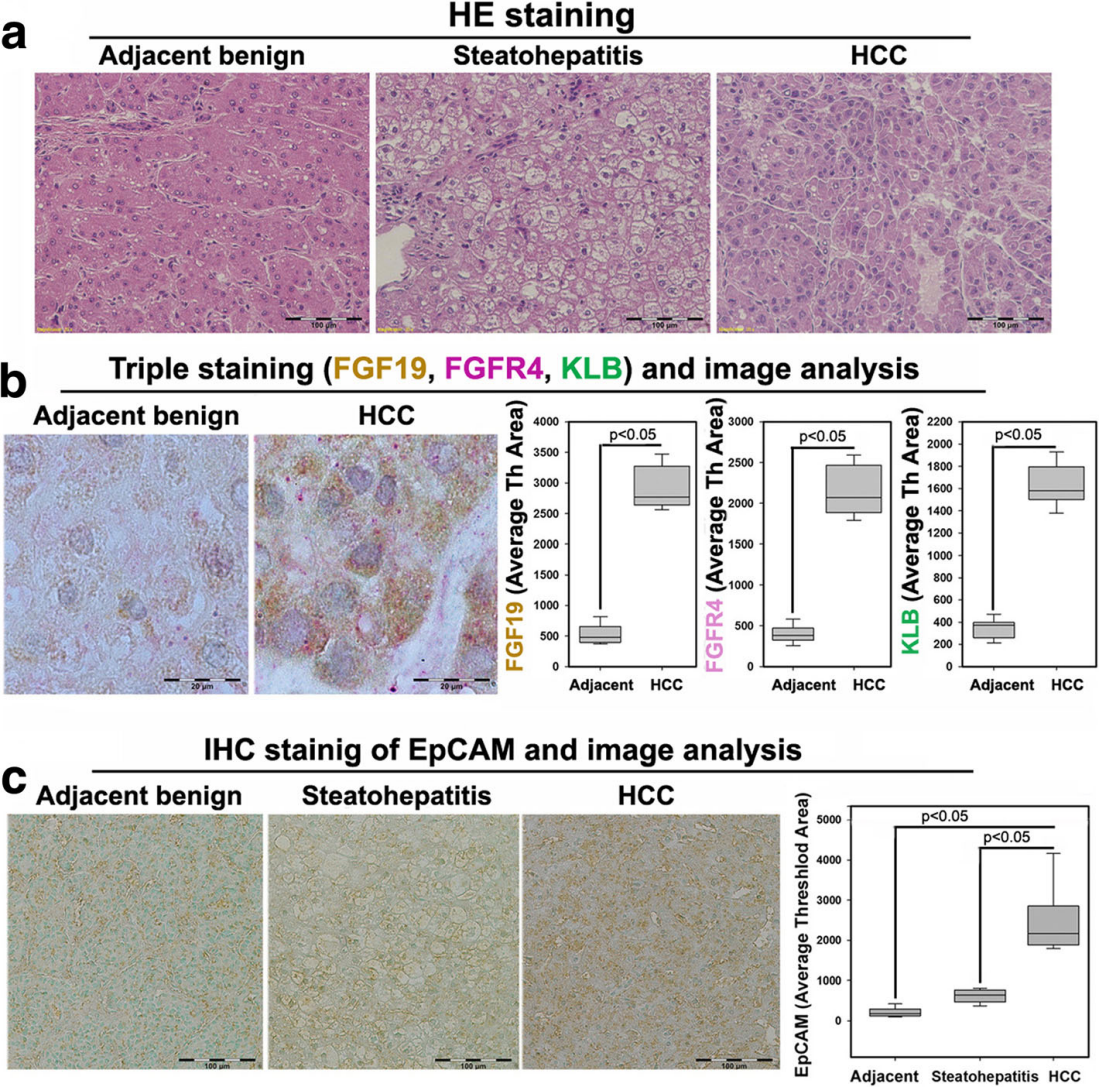
**a** Representative histology by H&E staining for HCC patients’ hepatic tissues including adjacent benign, steatohepatitis and HCC. **b** Representative triple staining for FGF19, FGFR4 and β-klotho in HCC patients’ hepatic tissues of adjacent benign and HCC. Brown color: FGF19; Pink color: FGFR4; Green color: β-klotho. The computer quantification of the expression was presented as Average of Threshold Area (Average Th Area). KLB: β-klotho; adjacent: adjacent benign. **c** Representative immunohistochemical staining of EpCAM and image analysis in HCC patients’ hepatic tissues including adjacent benign, steatohepatitis and HCC

The data from NASH-HCC mice suggested that aberrant FGF15/FGFR4 signaling and lipid metabolic disorder contributed to tumor-initiation for the carcinogenetic process. Further study for FGF19/FGFR4 signaling was performed in human HCC specimens with NASH background. The NASH-HCC pathologies were identified by H&E staining (Fig. 6a). Co-expression of FGF19/FGFR4/β-klotho was determined by triple IHC staining in the HCC tissues as well as in adjacent benign tissues. Expressions of FGF19/FGFR4/β-klotho were abundantly and simultaneously distributed in the HCC tissues. Image-analysis showed that there were significantly increased protein levels of FGF19/FGFR4/β-klotho, compared to the adjacent benign tissues (Fig. 6b). Significantly increased EpCAM expression was also observed in the in the HCC tissue, compared to the adjacent benign tissues (Fig. 6c). Consequently, the important components (FGFR4, FASN, AFP and β-catenin) related to lipid metabolic disorder, liver injury and CSC initiated the HCC transformation found in the animal studies; these were further evaluated by Western blot in the paired human samplers (tumor and benign) of 33 NASH-HCC patients. The results showed that protein levels of FGFR4, FASN, AFP and β-catenin were significantly up-regulated in malignant tissues compared to the adjacent benign tissues (Additional file 5: Figure S4).

## Discussion

The finding of FGF19 signaling contributing to HCC led to the speculation that FGF19 could be a promising molecular target for treatment of human HCC. In this study, we extended our previous human studies by reporting for the first time that FGF15 signaling plays a critical role in HCC initiation and development in a NASH-HCC mouse model. We found that hepatic FGF15/FGFR4 signaling is linked to EMT and Wnt/β-catenin pathways, contributing to the HCC carcinogenetic transformation.

As previously reported, FGF15/19 is not physiologically expressed in the liver [40], but pathological FGF19 expression in liver tissues was detected in the patients with hepatitis C virus cirrhosis and with biliary cirrhosis [41], which are closely related to HCC. It has been demonstrated that FGF19 and FFA can improve the steatotic mice’s outcome after hepatic resection, indicating FGF15/19 upon the mitogenic and cytoprotective effects is critical in protection of hetaptocyte from lipid-mediated cellular stress and injury [42]. The mitogenic and cytoprotective of FGF15/19 may protect the carcinogenetic transformed hepatocytes by DEN contributing to HCC in DEN+HFD mice. In our study, significantly increased FGF15 protein level was detected in “benign” and malignant liver tissues from DEN+HFD mice. The functional FGF15 signaling was further identified as the co-expression of FGF15/19-FGFR4-β-klotho in liver tissues from both HCC mice and HCC patients. Although FGF15/19 was a typical endocrine FGF, the extensive distribution of FGF15/19 protein levels from both mouse and human samples by IHC and the increased hepatic FGF15 mRNA expression from mouse samples suggested that FGF15/19 could function in an autocrine and paracrine fashion during HCC carcinogenetic transformation. The autocrine and paracrine fashions of FGF15 were supported by previous observations [29, 43, 44]. Importantly, in this study we found that the activated FGF15 signal positively linked to the pathways of EMT and Wnt/β-catenin signaling, which were well-accepted mechanisms for the tumor-initiation process. By using the FGFR4 inhibitor BLU9931, a robust reduction in the major components of Wnt/β-catenin and decreased migratory ability of HCC cells was found, indicating the FGF15/FGFR4-dependent activation of EMT and Wnt/β-catenin signaling. Our data was supported by previous reports in which FGF19 promotes EMT by modulating the GSK3β/β-catenin signaling cascade via FGFR4 activation [45, 46]. Our data strongly suggest that increased FGF15 signaling could mediate tumor cell initiation, thereby contributing to the HCC carcinogenesis. Although there was no previous report of an FGF15-linked tumor-initiation function, FGF15/19 was found as an important signal to stem cells for embryonic development [47]; for example, it is required for proper morphogenesis of the cardiac outflow tract [48]. Therefore, it is reasonable to speculate that FGF15 signaling mediated tumor-initiating carcinogenesis, but further study is needed.

Evidence reported in the literatures suggests that FGF19 is amplified in about 15% of human HCC tumors and is associated with a worse prognosis in HCC patients [27, 49, 50]. The most important finding form the current study is that the critical biomarkers contributing to carcinogenetic singling from NASH-HCC mice are also identified in the human HCC samples with NASH background, suggesting that the FGF19/FGFR4 axis might be a key driver in certain forms of HCC, such as NASH-HCC. In fact, pharmacological inhibition of FGF19/FGFR4 has been proven as efficacious experimental anti-HCC strategy [23, 51]. Supporting this view, we studied a specific FGFR4 inhibitor, BLU9931, which was well tested in selected HCC cell lines and HCC xenograft models [23] with tumoral FGF19/FGFR4/β-klotho expression. By using BLU9931, we found a robust reduction in these major components of EMT and Wnt/β-catenin signaling contributing to mice NASH-HCC. Silencing of the PPAR-α and CD36 genes significantly attenuated the FFA induced EpCAM, β-catenin and cyclinD1, further confirmed that FFA and FGF15/FGFR4 signaling could play a critical role in contributing to tumor-initiation and the development of HCC. Encouragingly, this study offers the advantages of the translational approach in mice and humans, and further effort is worthy and needed to study the FGF19/FGFR4 axis linked to tumor-initiation in human HCC.

Ectopic expression of FGF19 in skeletal muscle of transgenic mice elevated the hepatic AFP mRNA as early as 2 months of age, and the mice developed HCC at 10 months of age [52]. However, the role of FGF15 in carcinogenesis was under debate. Whereas the initial view of FGF15/19 was that of a key role in carcinogenetic transformation, a recent study noted that FGF19 and FGF15 exhibited a distinct profile with respect to the hepatocarcinogenesis [28]. It was found that FGF15, unlike FGF19, could not induce HCC in the mouse models of metabolic diseases, even at supra-pharmacological exposure levels [28]. Lack of an FGF15 knockout/knockdown model was the major limitation for both their study and our current study to address the hepatocarcinogenic role of FGF15.

## Conclusions

Up-regulated FGF15/FGFR4 signaling promoted the development of HCC by activation of EMT and Wnt/β-catenin signaling in the lipid metabolic disorder microenvironment. Further investigation of FGF19/FGFR4 signaling is important for potential early diagnosis and therapeutic targeting in HCC patients.

## Additional files

Additional file 1: **Tables S1-S3**, list of antibodies, promers and siRNAs. Method for ultrasound image acquisition. Figure legends for **Figures S1-S4**. (DOCX 18 kb)

Additional file 2: **Figure S1.** Representative gross anatomy and ultrasound images from all 4 experimental groups at month 2 month 6, and month 10. On visual pattern, tumor showed as HCC nodule, while the ultrasound appearance of HCC showed either to be hyperechoic or hypoechoic. M: month; UT: untreated; CD: control diet; HFD: high fat diet; DEN: N-nitrosodiethylamine. Black arrow head: HCC nodules on liver; White arrow head: HCC nodules on ultrasound images. (JPG 715 kb)

Additional file 3: **Figure S2.** The body weights, liver weights, serum and tissue triglyceride levels, alpha fetoprotein (AFP) and alanine transaminase (ALT) levels in all 4 experimental groups at month 2 month 6, and month 10. Glucose tolerance test (GTT) and insulin tolerance test (ITT) were recorded in all 4 experimental groups at month 10. UT: untreated; CD: control diet; HFD: high fat diet; DEN: N-nitrosodiethylamine. *: *P* < 0.05 vs UT + CD. (JPG 1775 kb)

Additional file 4: **Figure S3.** Representative images of EpCAM and β-Catenin in liver parenchyma in all 4 experimental groups at month 10 and in cultured cells treated with FFA and BAS. Fluorescent staining was performed using FITC tagged anti-EpCAM and anti-β-Catenin antibodies on the frozen tissue sections of mice. Fluorescent staining for HCC cells was carried out on the 8 well chamber slide seeded Hepal-6 cells in response to FFA treatment. DAPI (4′,6-diamidino-2-phenylindole) fluorescent stain was performed to detect nucleus as counter staining. UT: untreated; CD: control diet; HFD: high fat diet; DEN: N-nitrosodiethylamine; FFA: free fatty acid; BSA: bovine serum albumin. (JPG 4260 kb)

Additional file 5: **Figure S4.** Upper: Representative Western blot for β-Klotho proteins detection (AFP, FASN, FGFR4 and β-Catenin) of 3 paired tissues (HCC tissue and adjacent benign tissue) from HCC patients. Lower: quantification of AFP, FASN, FGFR4 and β-Catenin by Western blot analysis in tissues (HCC tissue and adjacent benign tissue) from 33 HCC patients. T: HCC tissue; A: adjacent benign tissue. *: P < 0.05 vs adjacent benign tissue. (JPG 608 kb)

## Abbreviations

AFP: Alpha-fetoprotein
ALT: Alanine aminotransferase
DEN: N-nitrosodiethylamine
EMT: Epithelial–mesenchymal transition
EpCAM: Epithelial cell adhesion molecule
FASN: Fatty acid synthase
FFA: Free fatty acids
FGF15/19: Fibroblast growth factor 15/19
FGFR4: Fibroblast growth factor receptor4
GTT: Glucose tolerance test
H&E taining: Hematoxylin-and-eosin staining
HCC: Hepatocellular carcinoma
HFD: High fat diet
IHC: Immunohistochemical
ITT: Insulin tolerance test
NAFLD: Non-alcoholic fatty liver disease
NASH: Nonalcoholic steatohepatitis
PPAR-α: Peroxisome proliferator-activated receptor α
