# Supplementary material for: Up-regulation of FGF15/19 signaling promotes hepatocellular carcinoma in the background of fatty liver
Source: J Exp Clin Cancer Res. 2018 Jul 4;37:136. doi: 10.1186/s13046-018-0781-8 (PMC6031179; doi:10.1186/s13046-018-0781-8)
Supplement: Supplementary file 1 — Tables S1-S3, list of antibodies, promers and siRNAs. Method for ultrasound image acquisition. Figure legends for Figures S1-S4. (DOCX 18 kb) [file 13046_2018_781_MOESM1_ESM.docx]

**Supplementary materials**

Table 1. List of antibodies for western blots and immunofluorescence staining.

| **Antibody** | **Source (catalog)** |
| --- | --- |
| FGF19 | Abcam (ab211431) |
| FGF15 | R&D (AF6755) |
| FGFR4 | Cell Signaling (#2894) |
| FAS | Santa Cruz (A-20) |
| β-catenin | Santa Cruz (E-5) |
| EpCAM | Sigma (SAB4200473 ) |
| Cyclin D1 | Cell Signaling (#2978) |
| E-cadherin | Cell Signaling (#14472) |
| Vimentin | Cell Signaling (#5741) |
| KLB | Abcam (ab106794) |
| AFP | Santa Cruz(H-140) |
| Anti-IgG-HRP | Santa Cruz (sc2020) |
| GAPDH | Santa Cruz(FL-335) |

Table 2. List of primers for RT-PCR

| **Primer** | **Gene ID** | **Sequence(5′->3′) mouse** | **Product length** |
| --- | --- | --- | --- |
| FGF15 | 14170 | Forward: 5′- GAGGACCAAAACGAACGAAATT-3′  Reverse: 5′- ACGTCCTTGATGGCAATCG-3′ | 71 |
| FGFR4 | 14186 | Forward: 5′- GCCTCCGACAAGGATTTGGCA -3′  Reverse: 5′- TGCAGACACCCAGCAGGT-3′ | 231 |
| IL-6 | 16193 | Forward: 5′-AAAGAGTTGTGCAATGGCAATTCT-3'  Reverse: 5′-AAGTGCATCATCGTTGTTCATACA-3' | 51 |
| TNF-α | 21926 | Forward: 5'-CTGTAGCCCACGTCGTAGC-3'  Reverse: 5'-TTGAGATCCATGCCGTTG-3' | 97 |
| TGF-β1 | 21803 | Forward: 5’-ATGTCACGGTTAGGGGCTC-3’  Reverse: 5’-GGCTTGCATACTGTGCTGTATAG-3’ | 146 |
| E-cadherin | 12550 | Forward: 5′- CACCTGGAGAGAGGCCATGT -3′  Reverse: 5′- TGGGAAACATGAGCAGCTCT -3′ | 298 |
| Vimentin | 22352 | Forward: 5′-CGGCTGCGAGAGAAATTGC-3′  Reverse: 5′-CCACTTTCCGTTCAAGGTCAAG-3′ | 124 |
| CD36 | 12491 | Forward: 5′- CATTTGCAGGTCTATCTACG -3′  Reverse: 5′- CAATGTCTAGCACACCATAAG -3′ | 162 |
| PPARα | 19013 | Forward: 5′- GATGTCACACAATGCAATTC -3′  Reverse: 5′- ACGTTTCCGAATCTTTCAGG -3′ | 88 |
| PPARγ | 19016 | Forward: 5′- AAAGACAACGGACAAATCAC-3′  Reverse: 5′- GGGATATTTTTGGCATACTCTG -3′ | 174 |

Table 3. List of siRNA

| **siRNA** | **Gene ID** | **Sequence(5′->3′) mouse** | **Product length** |
| --- | --- | --- | --- |
| **siPPARa (human)** | Hs.103110 | Sense (5'-3'): GAUCAAGUGACAUUGCUAAtt  Antisense (5'-3'): UAGCAAUGUCACUUGAUCgt | 21 |
| **siCD36**  **(human)** | Hs.173510 | Sense (5'-3'): CGACAUGAUUAAUGGUACAtt  Antisense (5'-3'): GUACCAUUAAUCAUGUCGca | 21 |
| **siPPARa (mouse)** | Mm.212789 | Sense (5'-3'): CGACCUGAAAGAUUCGGAAtt  Antisense (5'-3'): UUCCGAAUCUUUCAGGUCGtg | 21 |
| **siCD36**  **(mouse)** | Mm.18628 | Sense (5'-3'): CCACAUAUCUACCAAAAUUtt  Antisense (5'-3'): AAUUUUGGUAGAUAUGUGGtg | 21 |
| **Scrambled siRNA** | N/A | N/A | N/A |

**Method**

Ultrasound image acquisition

The animals were underwent ultrasound imaging monthly. All animals were food deprived for 12 hours before ultrasound. After induction and maintenance of anesthesia with 5% and 2% isoflurane, mice were shaved on abdomen, faced up and performed abdominal ultrasound examination. Ultrasound images were acquired by professional medical experts using a Visualsonics system (Vevo 2100, VisualSonics Inc, Canada) with bandwidth of MS250 and the fundamental frequency of 30 MHz. The contrast ratio and light intensity were set up as 60% and 50%, respectively. The dynamic range was fixed to 10 dB and the Time Gain Compensation was kept constant throughout the procedure. This study procedure was approved by the Institutional Animal Care and Use Committee at the University of Louisville.

**Figure legends**

**Figure S1:** Representative gross anatomy and ultrasound images from all 4 experimental groups at month 2 month 6, and month 10. On visual pattern, tumor showed as HCC nodule, while the ultrasound appearance of HCC showed either to be hyperechoic or hypoechoic. M: month; UT: untreated; CD: control diet; HFD: high fat diet; DEN: N-nitrosodiethylamine. Black arrow head: HCC nodules on liver; White arrow head: HCC nodules on ultrasound images.

**Figure S2:** The body weights, liver weights, serum and tissue triglyceride levels, alpha fetoprotein (AFP) and alanine transaminase (ALT) levels in all 4 experimental groups at month 2 month 6, and month 10. Glucose tolerance test (GTT) and insulin tolerance test (ITT) were recorded in all 4 experimental groups at month 10. UT: untreated; CD: control diet; HFD: high fat diet; DEN: N-nitrosodiethylamine. *: P<0.05 vs UT+CD.

**Figure S3:** Representative images of EpCAM and β-Catenin in liver parenchyma in all 4 experimental groups at month 10 and in cultured cells treated with FFA and BAS. Fluorescent staining was performed using FITC tagged anti-EpCAM and anti-β-Catenin antibodies on the frozen tissue sections of mice. Fluorescent staining for HCC cells was carried out on the 8 well chamber slide seeded Hepal-6 cells in response to FFA treatment. DAPI (4',6-diamidino-2-phenylindole) fluorescent stain was performed to detect nucleus as counter staining. UT: untreated; CD: control diet; HFD: high fat diet; DEN: N-nitrosodiethylamine; FFA: free fatty acid; BSA: bovine serum albumin.

**Figure S4:** Upper: Representative Western blot for β-Klotho proteins detection (AFP, FASN, FGFR4 and β-Catenin) of 3 paired tissues (HCC tissue and adjacent benign tissue) from HCC patients. Lower: quantification of AFP, FASN, FGFR4 and β-Catenin by Western blot analysis in tissues (HCC tissue and adjacent benign tissue) from 33 HCC patients. T: HCC tissue; A: adjacent benign tissue. *: P<0.05 vs adjacent benign tissue.
