# Supplementary figures and images for: Up-regulation of FGF15/19 signaling promotes hepatocellular carcinoma in the background of fatty liver
Source: J Exp Clin Cancer Res. 2018 Jul 4;37:136. doi: 10.1186/s13046-018-0781-8 (PMC6031179; doi:10.1186/s13046-018-0781-8)

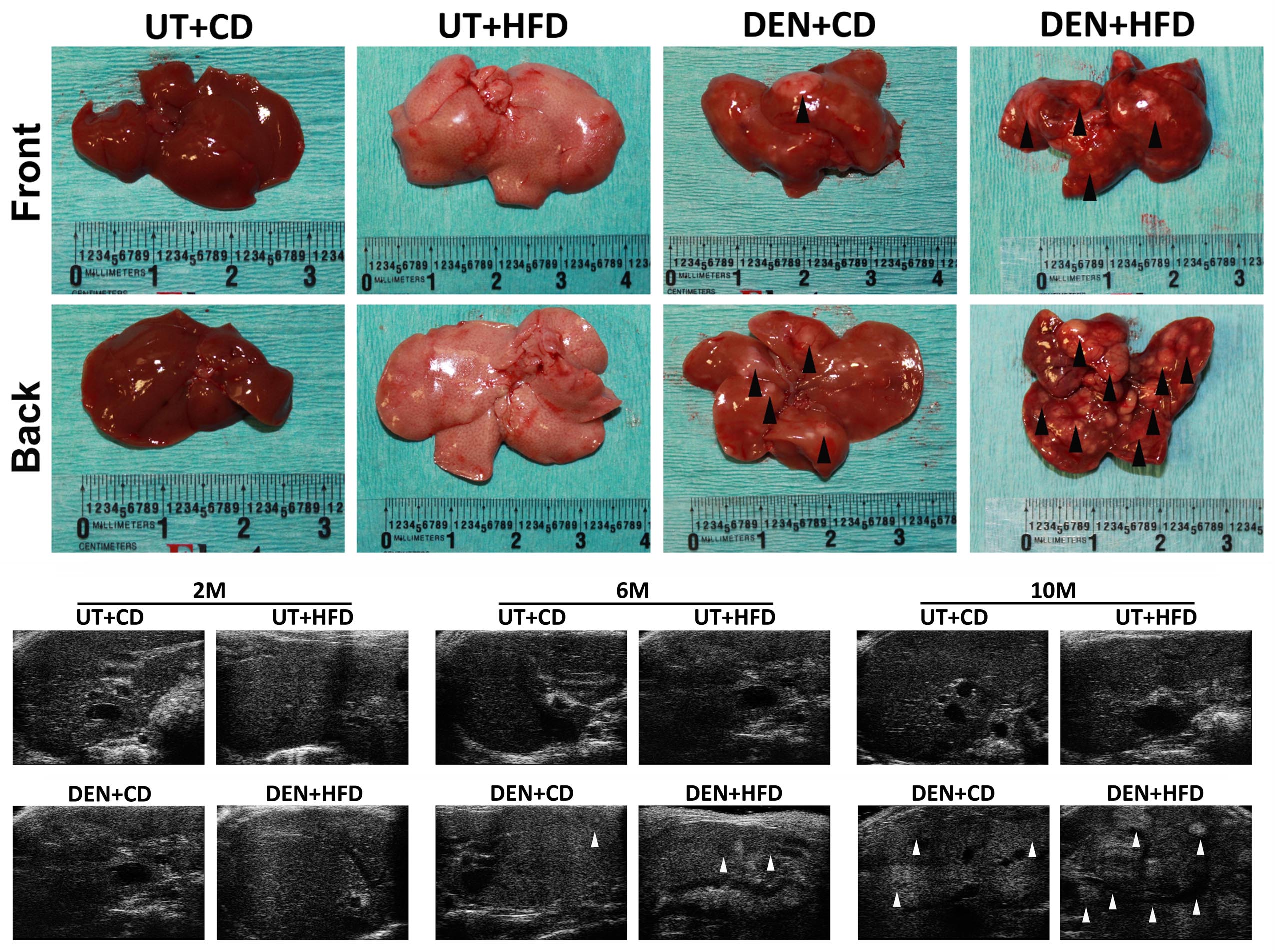

Supplement: Supplementary file 2 — Figure S1. Representative gross anatomy and ultrasound images from all 4 experimental groups at month 2 month 6, and month 10. On visual pattern, tumor showed as HCC nodule, while the ultrasound appearance of HCC showed either to be hyperechoic or hypoechoic. M: month; UT: untreated; CD: control diet; HFD: high fat diet; DEN: N-nitrosodiethylamine. Black arrow head: HCC nodules on liver; White arrow head: HCC nodules on ultrasound images. (JPG 715 kb) [file 13046_2018_781_MOESM2_ESM.jpg]

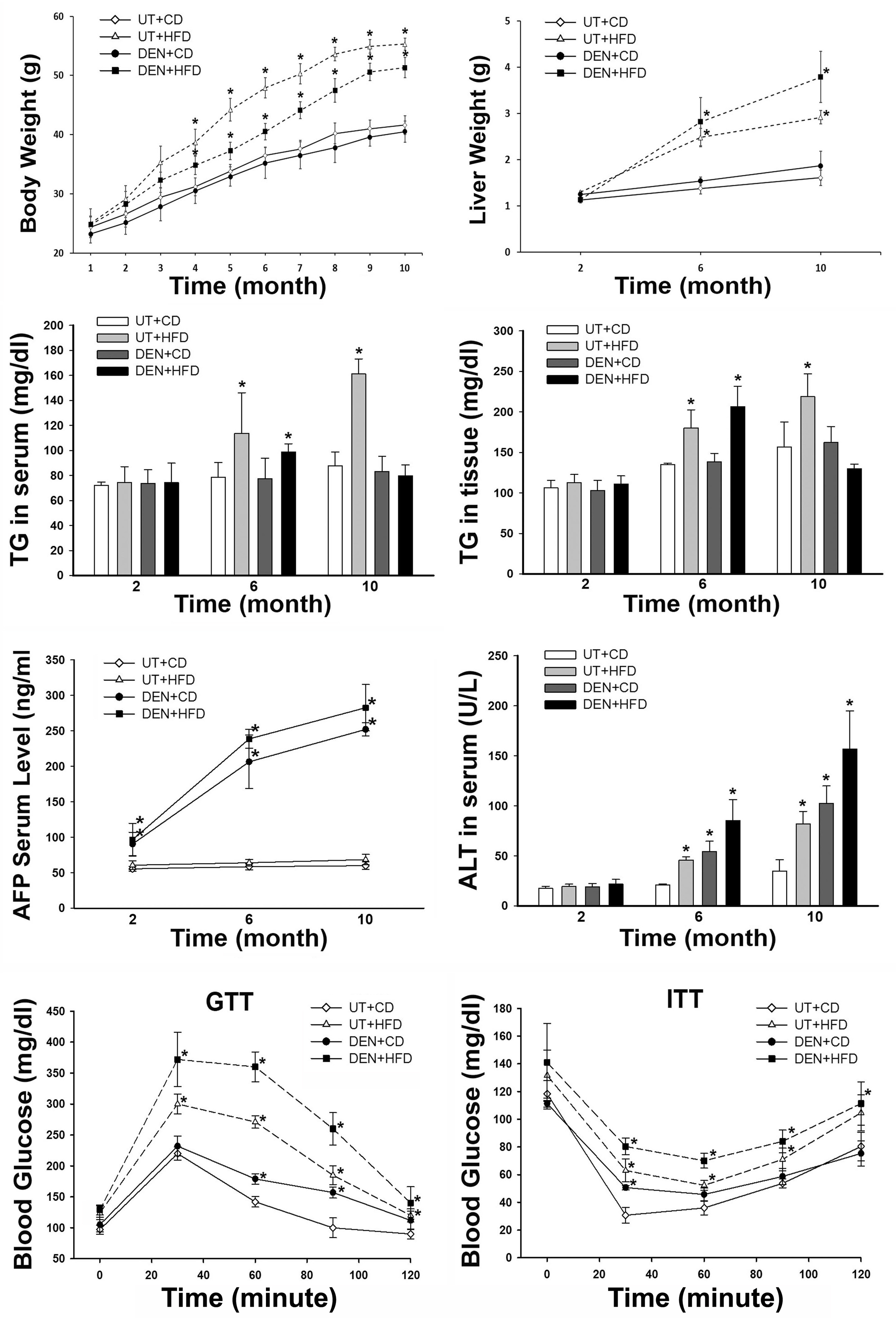

Supplement: Supplementary file 3 — Figure S2. The body weights, liver weights, serum and tissue triglyceride levels, alpha fetoprotein (AFP) and alanine transaminase (ALT) levels in all 4 experimental groups at month 2 month 6, and month 10. Glucose tolerance test (GTT) and insulin tolerance test (ITT) were recorded in all 4 experimental groups at month 10. UT: untreated; CD: control diet; HFD: high fat diet; DEN: N-nitrosodiethylamine. *: P < 0.05 vs UT + CD. (JPG 1775 kb) [file 13046_2018_781_MOESM3_ESM.jpg]

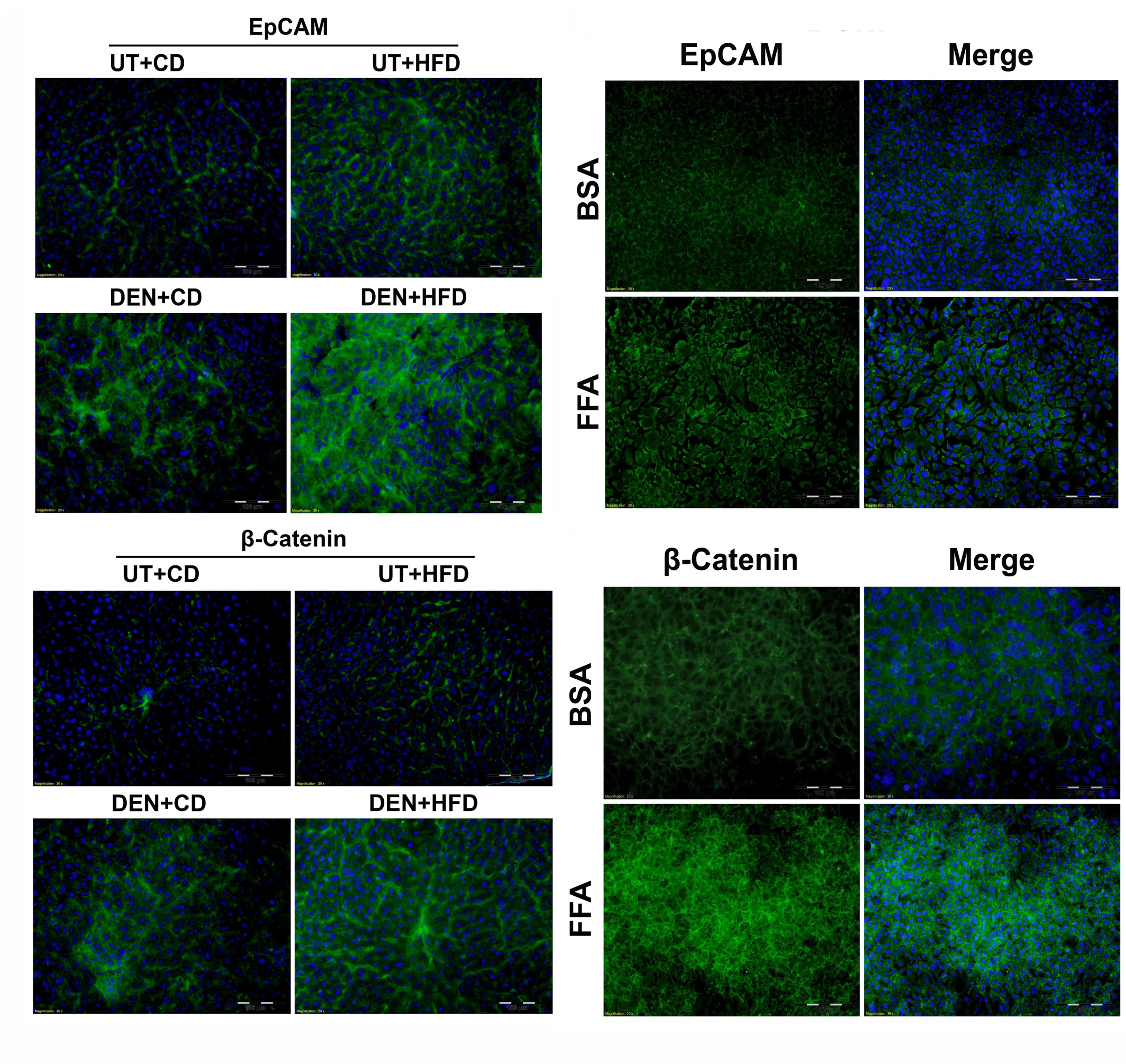

Supplement: Supplementary file 4 — Figure S3. Representative images of EpCAM and β-Catenin in liver parenchyma in all 4 experimental groups at month 10 and in cultured cells treated with FFA and BAS. Fluorescent staining was performed using FITC tagged anti-EpCAM and anti-β-Catenin antibodies on the frozen tissue sections of mice. Fluorescent staining for HCC cells was carried out on the 8 well chamber slide seeded Hepal-6 cells in response to FFA treatment. DAPI (4′,6-diamidino-2-phenylindole) fluorescent stain was performed to detect nucleus as counter staining. UT: untreated; CD: control diet; HFD: high fat diet; DEN: N-nitrosodiethylamine; FFA: free fatty acid; BSA: bovine serum albumin. (JPG 4260 kb) [file 13046_2018_781_MOESM4_ESM.jpg]

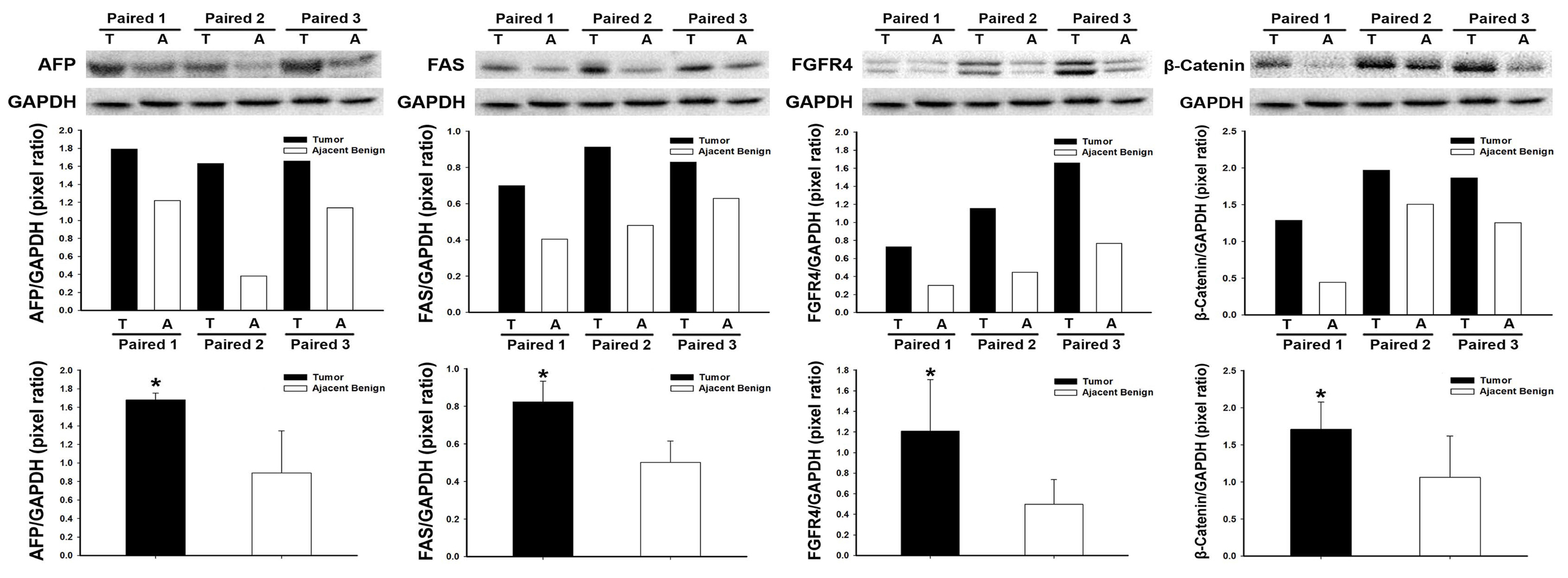

Supplement: Supplementary file 5 — Figure S4. Upper: Representative Western blot for β-Klotho proteins detection (AFP, FASN, FGFR4 and β-Catenin) of 3 paired tissues (HCC tissue and adjacent benign tissue) from HCC patients. Lower: quantification of AFP, FASN, FGFR4 and β-Catenin by Western blot analysis in tissues (HCC tissue and adjacent benign tissue) from 33 HCC patients. T: HCC tissue; A: adjacent benign tissue. *: P < 0.05 vs adjacent benign tissue. (JPG 608 kb) [file 13046_2018_781_MOESM5_ESM.jpg]
